# Antibiotics in neonatal life increase murine susceptibility to experimental psoriasis

**DOI:** 10.1038/ncomms9424

**Published:** 2015-09-29

**Authors:** Peter Zanvit, Joanne E. Konkel, Xue Jiao, Shimpei Kasagi, Dunfang Zhang, Ruiqing Wu, Cheryl Chia, Nadim J. Ajami, Daniel P. Smith, Joseph F. Petrosino, Brittany Abbatiello, Hiroko Nakatsukasa, Qianming Chen, Yasmine Belkaid, Zi-Jiang Chen, WanJun Chen

**Affiliations:** 1 Mucosal Immunology Section, OPCB, NIDCR, NIH, Bethesda, Maryland 20892, USA; 2 University of Manchester, Faculty of Life Sciences, Manchester, M13 9PT, UK; 3 Center of Reproductive Medicine, Shandong Provincial Hospital, Shandong University, Jinan 250001, China; 4 State Key Laboratory of Oral Diseases, West China Hospital of Stomatology, Sichuan University, Chengdu, Sichuan 610041, China; 5 The Alkek Center for Metagenomics and Microbiome Research, Department of Molecular Virology and Microbiology, Baylor College of Medicine, Houston, Texas 77030, USA; 6 Laboratory of Parasitic Diseases, NIAID, NIH, Bethesda, Maryland 20892, USA

## Abstract

Psoriasis is an inflammatory skin disease affecting ∼2% of the world's population, but the aetiology remains incompletely understood. Recently, microbiota have been shown to differentially regulate the development of autoimmune diseases, but their influence on psoriasis is incompletely understood. We show here that adult mice treated with antibiotics that target Gram-negative and Gram-positive bacteria develop ameliorated psoriasiform dermatitis induced by imiquimod, with decreased pro-inflammatory IL-17- and IL-22-producing T cells. Surprisingly, mice treated neonatally with these antibiotics develop exacerbated psoriasis induced by imiquimod or recombinant IL-23 injection when challenged as adults, with increased IL-22-producing γδ^+^ T cells. 16S rRNA gene compositional analysis reveals that neonatal antibiotic-treatment dysregulates gut and skin microbiota in adults, which is associated with increased susceptibility to experimental psoriasis. This link between neonatal antibiotic-mediated imbalance in microbiota and development of experimental psoriasis provides precedence for further investigation of its specific aetiology as it relates to human psoriasis.

## Abstract (web summary)

Commensal microbes are necessary for proper development of the immune system. Here Zanvit *et al*. show that neonatal antibiotics treatment causes long-term changes in the gut and skin microbiomes, and exacerbates immune-mediated skin pathology at adult age in mouse experimental models of psoriasis.

## Graphical abstract

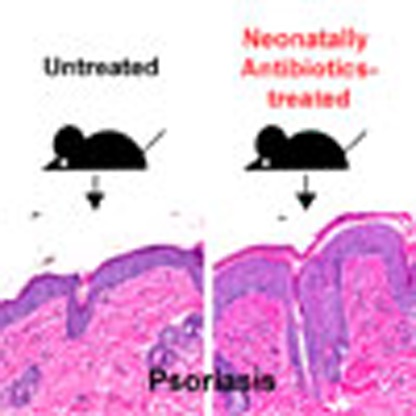

Psoriasis is an autoimmune-related chronic inflammatory disease with an active pathogenic interaction between the immune system and the skin^1^. Psoriasis affects ∼2% of the world's population, with increasing prevalence in developed countries^2^. This increase is thought to be triggered by a combination of genetic and environmental factors. The absence of 100% concordance in monozygotic twins and the lack of a clear inheritance pattern in families suggest a crucial role of environmental factors in disease development^3^. Accumulating evidence suggests that dysbiosis in the gut microbiota could play a critical role in the development of or predisposition for systemic immune diseases, such as rheumatoid arthritis^4^,^5^, encephalomyelitis^6^,^7^, type 1 diabetes^8^,^9^, and asthma^10^. However, the mechanism in which changes in microbial communities contribute to disease aetiology remains poorly defined. The human gut is colonized by 10^14^ bacteria, and contains upwards of 1,000 bacterial species^11^. Specific subsets of microbiota have been shown to differentially regulate immune function. *Bacteroides fragilis* via production of polysaccharide A can promote Tregs induction and control Th1/Th2 balance^12^,^13^, while Segmented filamentous bacteria direct Th17 cell differentiation^14^ and *Clostridium* species induce T_reg_ development^15^. However, whether gut microbiota influences the development and/or pathogenesis of psoriasis remains unknown.

Daily topical application of imiquimod (TLR7 agonist) on the skin and ears of mice leads to a psoriasis-like dermatitis with many hallmarks of human psoriasis including skin thickening, hyperkeratosis, acanthosis, scaling and erythema^16^. Development of disease in this model has been shown to be critically dependent on the IL-23/IL-17/IL-22 axis. By using this experimental model, we uncovered a different effect on experimental psoriasis in mice when we reduced gut microbiota in adult or neonatal mice with antibiotics. Antibiotic treated adult mice ameliorate imiquimod-induced psoriasis and exhibit reduced pro-inflammatory IL-17 and IL-22-producing T cells. Surprisingly, however, neonatally antibiotic-treated mice evoke exacerbated disease with significantly increased IL-22-producing γδ^+^ T cells, when psoriasis is induced in adult life.

## Results

### Imiquimod increases IL-17 and IL-22 in T cells in skin

**Figure 1:**
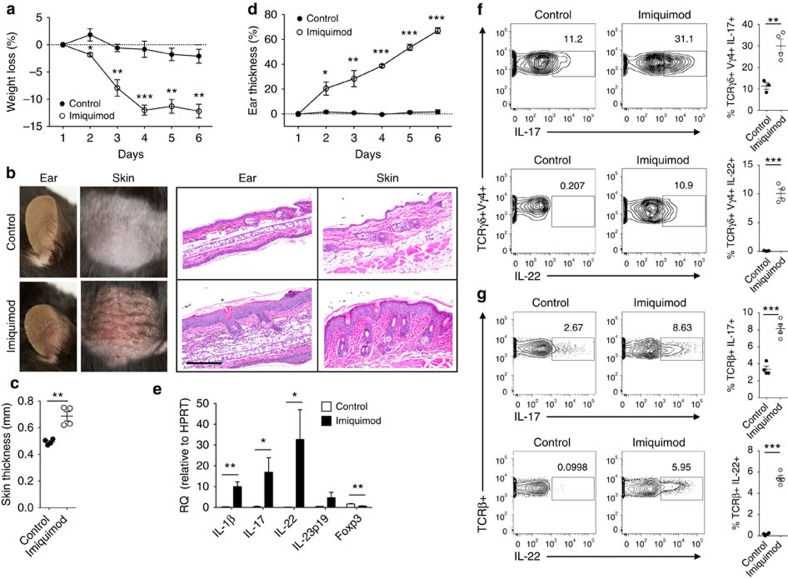
Imiquimod increases IL-17 and IL-22 in both γδ and αβ T cells in skin. Skin and ear of wild type C57BL/6 mice were treated by topical application of imiquimod cream (Fougera, *n*=4) or control cream (Vaseline, *n*=3) for 6 consecutive days. (**a**) Weight loss of imiquimod and control cream treated mice monitored daily. (**b**) Photographs of imiquimod and control cream treated skin and ear; photos were taken at day 6. H&E stained ear and back skin sections of imiquimod treated and control mice. Scale bar, 200 μm. (**c**) Thickness of skin measured by Digimatic Caliper at day 6 in control and imiquimod treated mice. Data showed represents average of at least two measurements. (**d**) Ear thickness of imiquimod and control cream treated mice monitored daily. Ear thickness was measured using Digimatic Caliper. (**e**) Quantitative PCR analysis of Th17 associated cytokines and Foxp3 in skin after 6 days of imiquimod and control cream treatment. (**f**) Representative flow cytometric analysis of TCRγδ^+^Vγ4^+^IL-17^+^ (upper raw) and TCRγδ^+^Vγ4^+^IL-22^+^ cells (lower row) in the skin of control (*n*=3) and imiquimod treated mice (*n*=4). (**g**) Representative flow cytometric analysis of single-positive TCRβ^+^IL-17^+^ (upper raw) or TCRβ^+^IL-22^+^ (lower raw) cells in the skin of control (*n*=3) and imiquimod treated mice (*n*=4). Data representative of more than three experiments, results are shown as mean±s.e.m., significance determined by unpaired two-tailed Student's *t*-test (*P<0.05; **P<0.01; ***P<0.001).

In mice, the application of imiquimod induces pathology that largely resembles the human psoriasis^16^,^17^. After 5–6 days of daily topical application of 5% imiquimod cream on skin and ears (Fougera), the mice showed significant weight loss (Fig. 1a), reddening and scaling of the skin (Fig. 1b) and thickening of skin (Fig. 1b,c) and ears (Fig. 1b,d). Imiquimod treatment resulted in hyperproliferation of keratinocytes and disturbed epidermal differentiation, as indicated by acanthosis and hyperparakeratosis (Fig. 1b). Dysregulated pro-inflammatory cytokines IL-17/23 and IL-22 responses are one of the hallmarks of psoriasis in rodents and human^1^,^3^. Imiquimod treatment resulted in significantly increased gene expression of *IL-17*, *IL-22* and *IL-1β* in skin (Fig. 1e). Flow cytometry analysis revealed that TCRβ^+^ and especially TCRγδ^+^Vγ4^+^ T cells (Heilig and Tonegawa nomenclature^18^) producing IL-17 and IL-22 were significantly increased in skin after imiquimod treatment (Fig. 1f,g, Supplementary Fig. 1a), consistent with recent reports^19^,^20^. The IL-17^+^ and IL-22^+^ γδ T cells were also increased in skin draining lymph nodes (dLNs) and Th17 and Th22 cells in spleen (Supplementary Fig. 1b,c), suggesting a systemic effect of imiquimod treatment on peripheral lymphoid tissues^16^. Thus, the data indicate that imiquimod-induced experimental psoriasis in mice shares many features of skin pathology and inflammatory pathogenesis of human psoriasis with increased IL-17 and IL-22 T cells.

### Antibiotic treatment of adult mice ameliorates psoriasis

**Figure 2:**
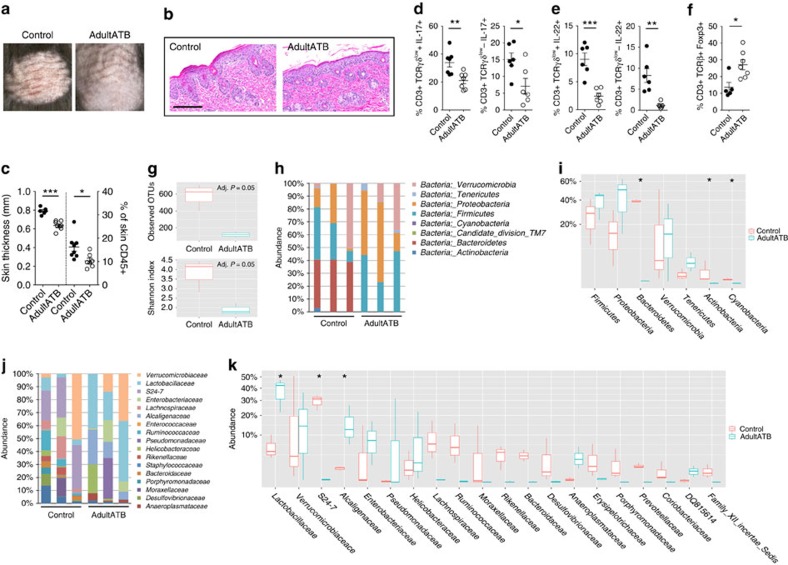
Antibiotics in adult mice ameliorate psoriasis by reduction of IL-17 and IL-22 in T cells. Young adult mice (4-weeks-old) were exposed to antibiotics in drinking water (adultATB) for four weeks, and last six consecutive days followed by imiquimod treatment. (**a**) Representative photographs of back skin of control and adultATB mice followed by imiquimod treatment. Photos were taken at day 6. (**b**) H&E stained skin sections of control and adultATB mice after imiquimod treatment. Scale bar, 200 μm. (**c**) Thickness of skin measured by digimatic caliper at day 6 in control and imiquimod treated mice. Data showed represents average of at least two measurements. Increased skin infiltration in control compared adultATB mice based on staining of skin CD45^+^ cells. (**d**) Flow cytometric analysis of single-positive CD3^+^TCRγδ^low+^IL-17^+^ cells or CD3^+^TCRγδ^−^IL-17^+^ cells in control (*n*=6–7) and adultATB (*n*=6–7) mice after imiquimod treatment. (**e**) Flow cytometric analysis of single-positive CD3^+^TCRγδ^low+^IL-22^+^ cells or CD3^+^TCRγδ^−^IL-22^+^ cells in control (*n*=6) and adultATB (*n*=6) mice after imiquimod treatment. (**f**) Frequency of skin CD3^+^TCRβ^+^Foxp3^+^ cells in the skin of control (*n*=5) and adultATB (*n*=7) mice after imiquimod treatment. (**g**) Diversity of the microbiota (Observed OTUs, Shannon Index) in adultATB and control mice. Primary samples were collected per cage (Pooled samples from 3 independent experiments) and analysed using 16S rRNA sequencing of region V4. (**h**) Relative abundance at phylum level between control and adultATB mice. Each column represents pooled samples from individual experiments. (**i**) Summarized phylum level analysis from three individual experiments showing difference between control and adultATB mice. (**j**) Relative abundance at family level between control and adultATB mice. Each column represents pooled samples from individual experiments. (**k**) Summarized family level analysis from three individual experiments showing difference between control and adultATB mice. Results are shown as mean±s.e.m., significance in (**c**–**f**) was determined by unpaired two-tailed Student's *t*-test (*P<0.05; **P<0.01; ***P<0.001), significance in (**i**,**k**) was determined using the non-parametric Kruskal–Wallis test (*P<0.05).

One of the major impacts of the mammalian microbiota is its effect on the development and function of the immune system. Recent studies in animal models have reinforced the notion that commensal microbiota influence systemic autoimmune diseases such as EAE, rheumatoid arthritis and asthma at sites distal to intestinal mucosa. Since specific subset of microbiota has been shown to play distinct roles in the development of Th17 cells (Segmented filamentous bacteria)^14^ or T_reg_ cells (*Clostridia* spp., *B. fragilis*)^12^,^13^,^15^, we hypothesized that commensal gut microbiota might regulate immune responses in the skin. For this, we treated mice with combination of antibiotics Vancomycin (targets G-positive bacteria) and Polymyxin B (targets G-negative bacteria) to deplete gut bacteria. Young adult mice (4-weeks-old) were exposed to antibiotics in drinking water for four weeks, and last 6 consecutive days followed by imiquimod treatment (Supplementary Fig. 2a). We observed that mice treated with antibiotics (AdultATB herein) showed decreased severity of psoriasiform dermatitis compared to mice that were not exposed to antibiotics (control) (Fig. 2a). Analysis of skin revealed significantly decreased thickness of skin and ears in mice exposed to antibiotics with decreased infiltration of CD45^+^ immune cells in skin (Fig. 2b,c, Supplementary Fig. 2b). We next investigated the underlying mechanisms responsible for the differential effects of the imiquimod-induced skin inflammation by antibiotics. We focused on IL-17^+^ and IL-22^+^ T cells as well as Foxp3^+^ T_reg_ cells in skin. We first analysed skin γδ T cells and observed surprisingly that the frequencies of IL-17^+^ γδ T cells (within CD3^+^TCRγδ^+low^) were significantly decreased in adultATB mice, compared with mice without antibiotic treatment (Fig. 2d). We found a similar effect of antibiotic treatment on expression of IL-17 by TCRαβ T cells (Fig. 2d). Furthermore, the frequencies of IL-22^+^ γδ T cells and IL-22^+^ αβ T cells were both significantly decreased in adultATB, compared with control mice without antibiotic treatment (Fig. 2e). The frequency of TCRβ^+^Foxp3^+^ cells was however significantly increased in the skin of adultATB mice (Fig. 2f). In the gut, we observed significant reduction of Th17 cells in lamina propria lymphocytes (LPL) of small intestine in adultATB mice compared with controls (Supplementary Fig. 2c), consistent with the profile of IL-17 in the skin (Fig. 2d). Decrease of Th17 cells corresponded to the decrease in the expression of CCR6 in TCRβ^+^CD4^+^ T cells in the gut; a chemokine receptor required for migration of gut Th17 cells to the skin inflammatory tissues^21^ (Supplementary Fig. 2d). Thus, we have demonstrated that the decrease in IL-22^+^ and IL-17^+^ T cells in the skin is likely a primary factor that accounts for the amelioration of the skin disease in mice depleted of gut bacteria.

In addition to depletion of gut bacteria, we also evaluated the effects of skin microflora depletion by direct skin topical application of antibiotics on imiquimod-induced psoriasis. Mice were treated by topical application of antibiotics (vancomycin+polymyxin B) on the skin (TopATB herein) followed by imiquimod treatment (see experimental scheme on Supplementary Fig. 3a). Treatment with antibiotic water significantly reduced skin microbiota (Supplementary Fig. 3b). We observed decreased acanthosis, decreased thickness of epidermis and decreased thickness of the skin in TopATB compared with untreated (control) mice (Supplementary Fig. 3c,d). Analysis of the skin T cells revealed that topical antibiotics treatment resulted in a decrease in Vγ4^+^IL-17^+^ and Vγ4^+^IL-22^+^ cells, but not Th17 and Th22 cells, compared with control mice (Supplementary Fig. 3d,e). In addition we have also utilized the treatment with antibiotic ointment for elimination of skin microbiota (Supplementary Fig. 4). Topical application of imiquimod in combination with Neosporin also effectively eliminated skin microbiota in the skin of TopATB mice (Supplementary Fig. 4). Importantly, we observed decreased severity of psoriasis-like disease in TopATB mice as indicated by decreased acanthosis, decreased thickness of skin and epidermis (Supplementary Fig. 4). Analyses of skin T cells revealed a decreased frequency of IL-22^+^ T cells in the skin of TopATB mice (Supplementary Fig. 4). The data altogether suggests that skin bacteria might be also involved in pathogenesis of imiquimod-induced psoriasis by affecting skin effector T cells.

### Microbiota analysis in mice given antibiotics in adult age

To investigate the global shift of gut bacterial communities caused by antibiotics treatment in adult mice, we have utilized sequencing of 16S rRNA from faecal samples collected after antibiotic and imiquimod treatment. We first employed alpha diversity metrics (observed operational taxonomic units (OTUs) and Shannon diversity index) to examine changes in commensal microbiota diversity between control and adultATB mice. The faeces of adultATB mice were found to have significantly lower Shannon diversity index values and fewer OTUs (Fig. 2g). Principal coordinate analysis (PCoA) plots derived from pairwise sample dissimilarity measures revealed significantly discrete clustering of the control and adultATB groups (Supplementary Fig. 2e). At the phylum level, we have observed decreased abundance of *Bacteroidetes* (Kruskal–Wallis test, *P*=0.04953), *Actinobacteria* (Kruskal–Wallis test, *P*=0.0463) and *Cyanobacteria* (Kruskal–Wallis test, *P*=0.0369) in adultATB mice (Fig. 2h,i). At the family level, *Lactobacillaceae* (Kruskal–Wallis test, *P*=0.04953) and *Alcaligenaceae* (Kruskal–Wallis test, *P*=0.04953) represented significantly increased families in adultATB mice, whereas the *Bacteroidales* S24-7 (Kruskal–Wallis test, *P*=0.04953) family was significantly decreased in adultATB mice (Fig. 2j,k). We also analysed the changes in the skin microbiota by 16S rRNA sequencing analysis, using skin swabs collected from adultATB and control mice before and after imiquimod treatment. Before imiquimod application, adultATB mice exhibited lower diversity in their skin microbiome, as measured by the number of observed OTUs. After imiquimod treatment, no difference was observed between the adultATB and control groups (Supplementary Fig. 5a,d). Abundances of phyla were consistent between treatment groups both before and after imiquimod treatment (Supplementary Fig. 5b,e). Moreover we identified top 25 different taxa on the skin of control or adultATB mice before and after imiquimod treatment (Supplementary Fig. 5c,f), however, because of extremely high variability of the skin commensals, we were not able to identify any significant differences between adultATB and control mice. On the other hand, high variability in the skin commensals could result from passive transfer of antibiotic water or faecal microflora within the cage during housing of mice.

### Neonatal antibiotic treatment increases psoriasis severity

**Figure 3:**
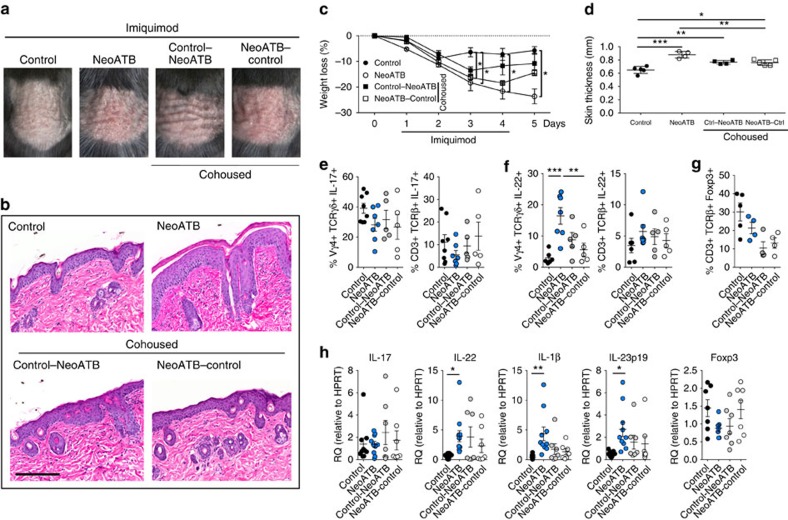
Neonatal antibiotic treatment of mice increases severity of psoriasis. Breeding pairs were given antibiotics vancomycin+polymyxin B in drinking water immediately after pups delivery for 3 weeks of rearing (neoATB). After weaning, mice were housed or cohoused (mice without antibiotics treatment together with mice treated with antibiotics) in normal specific pathogen-free conditions for next 8 weeks. The mice were then treated with imiquimod cream for 4 consecutive days. (**a**) Representative photographs of back skin in controls (mice without antibiotic treatment); neonatally antibiotics treated mice (neoATB) or cohoused mice (control–neoATB resp. NeoATB–control) after imiquimod treatment. Photos were taken at day 5. (**b**) H&E stained skin sections of control, neoATB and cohoused mice after imiquimod treatment. Scale bar, 200 μm. (**c**) Weight loss of control, neoATB and cohoused mice during imiquimod treatment monitored daily. (**d**) Skin thickness of control, neoATB and cohoused mice measured at day 5. Skin thickness was measured by digimatic caliper by at least two investigators and data showed represents average of at least two measurements. (**e**) Flow cytometric analysis of single-positive TCRγδ^+^Vγ4^+^IL-17^+^ cells or CD3^+^TCRβ^+^ IL-17^+^ cells in control (*n*=8), neoATB (*n*=7) and cohoused mice (*n*=5) after imiquimod treatment. (**f**) Flow cytometric analysis of single-positive TCRγδ^+^Vγ4^+^IL-22^+^ cells or CD3^+^TCRβ^+^ IL-22^+^ cells in control (*n*=6), neoATB (*n*=7) and cohoused mice (*n*=5) after imiquimod treatment. (**g**) Frequency of skin TCRβ^+^CD3^+^Foxp3^+^ cells in control (*n*=5), neoATB (*n*=4) and cohoused mice (*n*=4) after imiquimod treatment. (**h**) Quantitative PCR analysis of IL-17, IL-22, IL-1β, IL-23p19 and Foxp3 in the skin of control (*n*=7–10), neoATB (*n*=7–10) and Cohoused mice (*n*=7) after imiquimod treatment. Data representative of five experiments (control versus neoATB) or two experiments (Cohoused), results are shown as mean±s.e.m., significance in (**c**) was determined by one-way analysis of variance followed by Dunnett posttest or in (**d**–**h**) by Tukey's posttest (*P<0.05; **P<0.01; ***P<0.001).

At the time of birth, the host's immune system is immature. Compelling evidence from human and mouse studies suggests that colonization of the gut early in life has a substantial role in directing immune system development^22^. It has been shown that in humans the main bacterial populations comprising microbiota stabilize during the first 2–3 years of life^23^,^24^. During this time, the microbiota develops and subsequently remains stable throughout the life. Dysregulation of microbiota, for example, through antibiotic use during infancy has been linked with increased risk of development of allergy and asthma and even obesity later in life^10^,^25^,^26^. We wanted to determine the effect of early microbial colonization on the development of imiquimod-induced psoriasis. To this end, breeding pairs were given antibiotics in drinking water (vancomycin+polymyxin B) immediately after delivery of pups for 3 weeks of rearing. After weaning, mice were housed or cohoused (untreated mice together with antibiotics treated mice) in normal specific pathogen-free conditions for next 8 weeks. The mice were then treated with imiquimod cream for 4 consecutive days (experimental scheme see Supplementary Fig. 6a). After imiquimod treatment we observed, surprisingly, increased severity of disease, increased skin thickness and weight loss in mice neonatally treated with antibiotics (neoATBs) compared with mice without antibiotic treatment (control) (Fig. 3a–c). Histological analysis of skin biopsies revealed increased thickness of the skin with dermal infiltrate in neoATB mice (Fig. 3d).

To understand the underlying mechanisms responsible for the exacerbation of skin psoriasis in mice treated with antibiotics neonatally, we investigated the inflammatory cytokines IL-17 and IL-22 in skin T cells. Surprisingly, we did not find increases in IL-17^+^ cells in either αβ^+^ or γδ^+^ T cells (Fig. 3e). In fact, frequencies of IL-17^+^ T cell, and in particular, TCRαβ^+^ IL-17^+^ cells decreased (albeit no statistical significance) in the skin of neoATB mice compared to untreated control mice (Fig. 3e). Similar trends of decreased frequencies of Th17^+^ cells were also observed in the gut, skin dLNs and spleens of neoATB mice (Supplementary Fig. 6b,c). Strikingly, and in marked contrast, we found that neoATB-treatment led to a significant increase in TCRγδ^low+^Vγ4^+^ IL-22^+^ cells in the skin compared to untreated control mice (Fig. 3f). The frequency of TCRαβ^+^IL-22^+^ T cells was also higher, but did not reach statistical significance (Fig. 3f). NeoATB mice showed no significant changes of the frequency of Foxp3^+^ T cells in skin compared with control mice after imiquimod exposure (Fig. 3g). The frequencies of Foxp3^+^ cells in the dLNs, spleen and gut were not significantly changed in neoATB mice compared with controls (Supplementary Fig. 6c,d). Consistent with the intracellular cytokine data, qPCR analysis revealed significantly increased gene expression of *IL-22*, *IL-1β* and *IL-23*, but not *IL-17* in neoATB mice compared to control mice (Fig. 3h). To confirm the role of IL-22 in the exacerbated psoriasis in neoATB mice, we intradermally injected anti-IL-22 (neoATB+αIL-22) or isotype control antibodies into neoATB mice at days 1 and 3 during imiquimod treatment (see treatment scheme on Supplementary Fig. 7a). As expected, an increased severity of disease together with significantly increased skin and epidermis thickness was observed in neoATB+IgG treated compared with control treated mice (Supplementary Fig. 7b–e). However, anti-IL-22 antibody (neoATB+αIL-22) treatment significantly reduced the severity of the disease and skin thickness (Supplementary Fig. 7b–e). There was no effect of IL-22 neutralization on the weight loss in neoATB mice, suggesting a local effect of anti-IL-22 antibody (Supplementary Fig. 7f). Taken altogether, we demonstrate that increased IL-22-producing T cells play a critical role in the development of exacerbated disease in neoATB mice. Thus, neonatal elimination of gut microbiota increases the susceptibility to imiquimod-induced psoriasis in adults, which is likely attributed to the increase in IL-22-producing T cells, especially γδ^+^ T cells.

### IL-23 exacerbates psoriasis in neonatally treated mice

To further strengthen the conclusions that dysregulation of microbiota in neonatal age affects skin inflammation derived from imiquimod model, we have utilized another model of psoriasis based on intradermal injection of recombinant IL-23 (ref. 27). Control or neoATB mice (same protocol as in Supplementary Fig. 6a) were intradermally injected with recombinant IL-23 (1 μg per day) in two locations (0.5 μg to each) on either side of shaved back skin for 6 consecutive days (immunization scheme on Supplementary Fig. 8a). In this model, we showed that neoATB mice developed a more severe form of skin disease (Supplementary Fig. 8b). Importantly, as shown in the imiquimod model, we found that intradermal injection of rIL-23 in neoATB mice induced a significant increase in the frequency of TCRγδ^+^Vγ4^+^IL-22^+^ cells, but also induced a decrease in the frequency of TCRγδ^+^Vγ4^+^IL-17^+^ cells (Supplementary Fig. 8c,d). We did not observe significant differences in the production of IL-17 or IL-22 from TCRβ^+^ cells (Supplementary Fig. 8e,f). Thus, neoATB mice also exhibit more exacerbated skin disease in this IL-23-induced psoriasis model, which is again likely attributed to the increased IL-22-producing TCRγδ^+^Vγ4^+^ cells.

### Cohousing neonatally treated mice with controls improves disease

To test if transfer of ‘healthy' microbiota can overcome the effects of microbiota dysbiosis in neonatally antibiotic treated mice, we cohoused control mice together with neonatally antibiotics treated mice immediately after weaning for 8 weeks before imiquimod application (see scheme, Supplementary Fig. 6a). NeoATB mice that were cohoused with control mice (neoATB–control) developed a milder form of disease with less weight loss and decreased thickness of skin compared to neoATB mice (Fig. 3a–d). These cohoused neoATB–control mice exhibited slight but reproducible increases in frequencies of Th17 and TCRγδ^+^ IL-17^+^ cells in the skin, but showed a significant decrease in frequencies of TCRγδ^+^Vγ4^+^IL-22^+^ cells compared with neoATB mice (Fig. 3e,f). In the gut we also detected increased frequencies of Th17 cells in the cohoused neoATB–control mice compared with neoATB mice (Supplementary Fig. 6b). The frequencies of TCRβ^+^Foxp3^+^ T cells in neoATB–control mice were not significantly changed in the skin (Fig. 3g), in peripheral lymphoid tissues or in the gut (Supplementary Fig. 6c,d). In contrast, mice without antibiotic treatment cohoused with neoATB mice (control–neoATB) showed more severe skin inflammation and increased thickness of skin compared to controls alone (control) (Fig. 3a–d). Interestingly, control–neoATB mice had increased frequencies of TCRγδ^+^IL-22^+^ cells in the skin compared with control mice, albeit with no statistical significance (Fig. 3f). Frequencies of Th17 cells did not change significantly (Fig. 3e). In the gut, the frequency of Th17 cells was substantially increased in control–neoATB mice compared to neoATB mice (Supplementary Fig. 6b). The frequencies of TCRβ^+^CD3^+^Foxp3^+^ cells were slightly decreased in control–neoATB mice compared with control mice (Fig. 3g).

Taken altogether, we have revealed a previously unrecognized role of gut commensal microbiota on the development of skin psoriatic inflammation induced by either imiquimod or by intradermal injection of IL-23. The increased IL-22-producing T cells in the skin are likely the major inflammatory cells for the exacerbation of the disease. Here we have shown that early ‘correct' colonization of gut with commensal bacteria can prevent development of the severe form of psoriasis. Transplantation of ‘healthy' microbiota by cohousing the neoATB mice together with normal control mice could reduce the skin inflammation in the adult age, likely by re-balancing the dysregulated microbiota caused by antibiotics treatment early in life.

### Microbial dysbiosis in neonatally treated mice in adulthood

**Figure 4:**
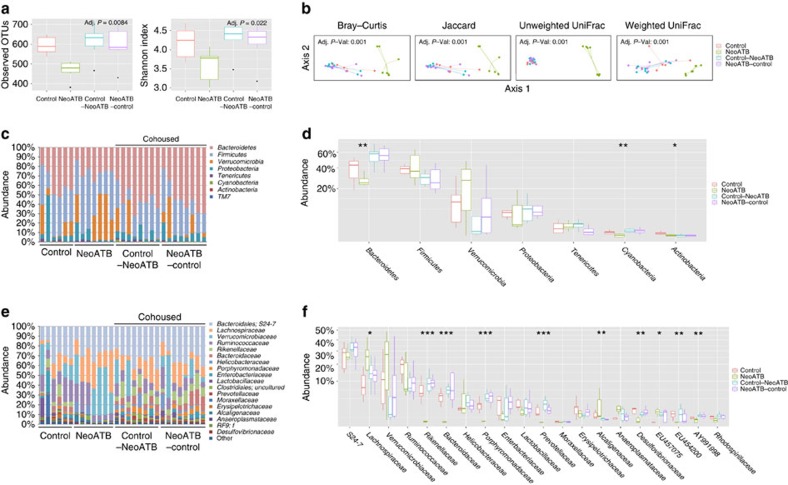
Neonatal antibiotic treatment alters bacterial communities in adult age. Faecal samples were collected per mouse and analysed by 16S rRNA sequencing of region V4. (**a**) Diversity of the microbiota (Observed OTUs, Shannon Index) in control (*n*=6), neoATB (*n*=7), control–neoATB (*n*=8) and neoATB–control (*n*=8) mice. (**b**) Beta diversity (PCoA) was used to compare distance measure (Unweighted and Weighted Unifrac), diversity (Jaccard) or dissimilarity (Bray–Curtis) between control, neoATB, control–neoATB and neoATB–control treatments. Relative abundance at Phylum (**c**,**d**) or Family (**e**,**f**) levels between control, neoATB, control–neoATB and neoATB–control mice. Each column in (**c**,**e**) represents individual mouse. (**d**–**f**) represents summary data from two individual experiments at phylum (**d**) or family (**f**) level between control, neoATB, control–neoATB and neoATB–control mice. Significance was determined using the non-parametric Kruskal–Wallis test (*P<0.05; **P<0.01; ***P<0.001).

To determine that the gut bacterial communities were indeed dysregulated in mice with neonatal antibiotic treatment, we analysed gut microbiota using 16S rRNA gene sequencing analysis in the aforementioned four groups: control, neoATB, control–neoATB and neoATB–control. Faeces of neoATB mice contained significantly less complex bacterial community than the other treatment groups, quantified by number of observed OTUs and Shannon's alpha diversity index (Fig. 4a). Similarity between sample microbiomes was calculated with the Bray–Curtis; Jaccard; weighted normalized UniFrac, and unweighted UniFrac formulas, then ordinated in two-dimensional space using PCoA. These plots confirm that faecal microbiomes of neoATB mice are distinct from control and cohoused mice (Fig. 4b). In faeces of neoATB treated mice, we observed significantly decreased abundances of the phylums *Bacteroidetes* (Kruskal–Wallis test, *P*=0.0038)*, Cyanobacteria* (Kruskal–Wallis test, *P*=0.0028) and *Actinobacteria* (Kruskal–Wallis test, *P*=0.035) (Fig. 4c,d). At the family level, *Lachnospiraceae* (Kruskal–Wallis test, *P*=0.0168) and *Alcaligenaceae* (Kruskal–Wallis test, *P*=0.003) represented significantly increased families in neoATB mice compared to control and cohoused mice. We also observed significantly decreased abundance of families *Rikenellaceae* (Kruskal–Wallis test, *P*=0.0006)*, Bacteroidaceae* (Kruskal–Wallis test, *P*=0.0006), *Porphyromonadaceae* (Kruskal–Wallis test, *P*=0.0002), *Prevotellaceae* (Kruskal–Wallis test, *P*=0.0002), *Desulfovibrionaceae* (Kruskal–Wallis test, *P*=0.006), and clades identified by the SILVA database entries *EU457075* (Kruskal–Wallis test, *P*=0.0146), *EU454200* (Kruskal–Wallis test, *P*=0.006) and *AY991998* (Kruskal–Wallis test, *P*=0.002) in neoATB compared with control and cohoused mice (Fig. 4d). Collectively, these data show that antibiotic treatment in neonatal age dysregulated the stable formation and diversity of gut microbiota compared to untreated controls. Intriguingly, in neoATB treated mice, commensal microbiota still remained dysregulated in adult age even when the mice were exposed to the normal environment for additional 8 weeks after the antibiotics were stopped and should have had a chance to reacquire normal microbiota (Fig. 4). Importantly, the dysbiosis of the neoATB mice can be corrected by cohousing early enough with control mice.

In addition to gut microbiota, we also compared the skin microbiota between neoATB and control mice in adult age before and after imiquimod treatment utilizing 16S rRNA sequencing analysis. Alpha diversity metrics revealed slightly decreased number of observed OTUs in the skin of neoATB treated mice before, but not after imiquimod treatment, when compared with control mice (Supplementary Fig. 9a,d). We did not observe significant differences at the phylum level in skin microbiota between neoATB and control mice (Supplementary Fig. 9b,c). Although data from the imiquimod treatment suggest an increase of some bacteria in the skin of neoATB mice, such as *Fusobacteria* (Supplementary Fig. 9e,f), the extremely high variability of the skin commensals precluded us from identifying any particular taxa significantly different between neoATB and control mice (Supplementary Fig. 9).

### Microbiota change between neonatally and adult-treated mice

**Figure 5:**
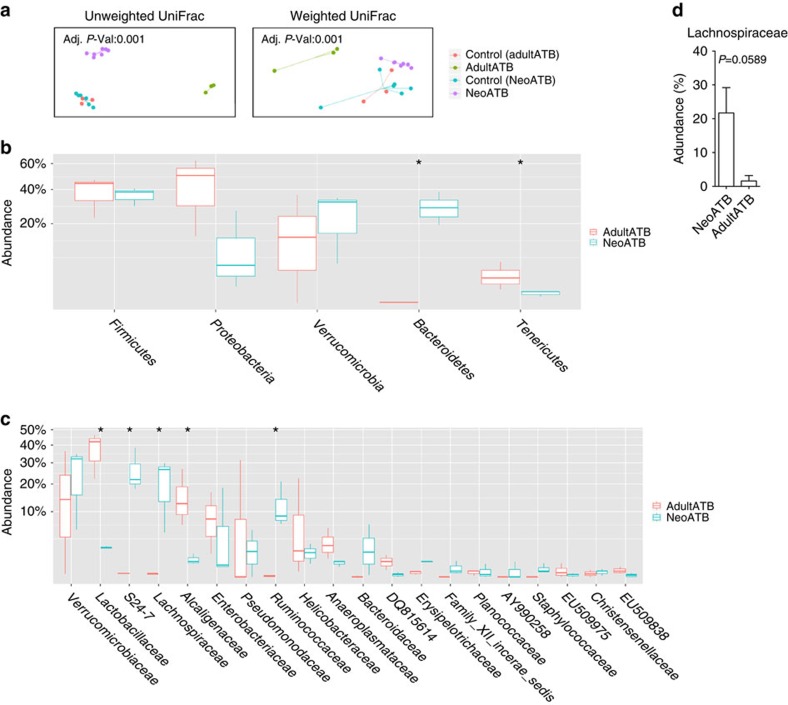
Changes in gut microflora composition after antibiotic treatment in adult versus neonatal antibiotic treated adult mice. (**a**) PCoA bi-plot illustrating the clustering of samples based on unweighted and weighted Unifrac distances between adultATB and neoATB treated mice and their controls. (**b**) Phylum level comparison of gut commensal microflora between adultATB and adult neoATB mice. (**c**) Family level comparison of gut commensal microflora between adultATB and adult neoATB mice. (**d**) Increased abundance of family *Lachnospiraceae* on the skin of neoATB compared with adultATB mice. Data are representative of two independent neoATB and three independent adultATB experiments. Significance in (**b**,**c**) was determined using the non-parametric Kruskal–Wallis test (*P<0.05).

We next identified specific taxa that correlated with functional outcomes among adultATB and neoATB mice, when mice reached adult age following imiquimod treatment. PCoA plots of unweighted and weighted UniFrac distances between samples revealed significant ordinations of both neoATB and adultATB distinct from one another and the control groups (Fig. 5a). At the phylum level, we have identified significantly decreased abundance of *Bacteroidetes* (Kruskal–Wallis test, *P*=0.04953) and increased abundance of *Tenericutes* (Kruskal–Wallis test, *P*=0.04953) in adultATB compared with neoATB mice (Fig. 5b). At the family level, major contributors to the observed disease outcomes are more abundant *Lactobacillaceae* (Kruskal–Wallis test, *P*=0.04953) and *Alcaligenaceae* (Kruskal–Wallis test, *P*=0.04953) families in adultATB mice compared with neoATB mice, whereas families *S24-7* (Kruskal–Wallis test, *P*=0.04953), *Lachnospiraceae* (Kruskal–Wallis test, *P*=0.04953) and *Ruminococcaceae* (Kruskal–Wallis test, *P*=0.04953) were significantly higher in neoATB mice compared to adultATB mice (Fig. 5c). We have also observed increased abundance of family *Lachnospiraceae* not only in the gut but also on the skin of neoATB compared to adultATB mice (Fig. 5d). The data suggest possible function of the changes in the aforementioned microbiota for the differential susceptibility to skin inflammation between neonatal and adult depletion of gut bacteria, but the causative relationship remains to be elucidated.

## Discussion

Psoriasis is an autoimmune-related chronic inflammatory disease with an active interaction between the immune system and the skin with unknown aetiology. It has been demonstrated that repeated topical application of imiquimod cream in mice represents a valuable experimental model for human psoriasis^16^. Effector T cells that produce IL-17 and IL-22 (including γδ T cells and CD4^+^ Th17 and Th22 cells), have a major role in psoriatic inflammation^19^,^20^. In addition to IL-17, IL-22 has been recently shown to be a critical driver of imiquimod-induced cutaneous inflammation^28^. IL-22-deficient mice, or mice treated with IL-22 neutralizing antibodies were protected against psoriasis induced by imiquimod. Here we confirm that skin TCRγδ^+^ cells are a major source of IL-17 and IL-22. Importantly, we identified the Vγ4^+^TCRγδ^+^ population in particular as the major cellular source of IL-17/IL-22 in skin under inflammatory conditions. Intriguingly, and as noted in our experiments and other published work, weight loss of ∼15% was observed in C57BL/6 mice treated with imiquimod, which was suggested to be due to pyrogenic response and transiently elevated IL-6 (ref. 16). However, these effects have not been documented in BALB/c mice and are likely to be strain specific. Despite this difference in weight loss, both mouse strains are able to develop psoriasis on imiquimod treatment^16^.

The aetiology of psoriasis still remains unknown, but both genetic and environmental factors seem to play important roles. The colonization of the gut begins at birth and is characterized by a succession of microbial consortia, the composition of which is influenced by changes in diet and by life events. Thus, mammalian health is absolutely dependent on microbial symbiosis. We have demonstrated here, that antibiotics-mediated depletion of gut microflora in adult age in fact ameliorates severity of psoriasis induced by imiquimod, together with decreased production of IL-17 and IL-22 cytokines by skin T cells. Adult age antibiotic treatment changed not only microflora composition in gut, but also on the skin. Furthermore, we have shown that reduction of skin commensal bacteria by topical antibiotic treatment might also decrease the severity of disease induced by imiquimod by reduction of IL-17- and IL-22- producing cells.

Infancy represents a time point when key immunological thresholds are established. Dysregulation of microbiota, for example, through antibiotic use during infancy has been linked with increased risk of development of allergy and asthma and even obesity later in life^10^,^25^,^26^. We show here that neonatal perturbation of gut microbiota with antibiotics treatment in mice increases the susceptibility and the severity of psoriasis induced by imiquimod later in adults. Surprisingly, the exacerbation of the skin inflammation is not associated with the increase in IL-17-prodcuing αβ^+^ or γδ^+^ T cells. Instead, the increased severity of skin inflammation was mainly attributed to significant increase in skin IL-22-producing T cells especially TCRγδ^+^IL-22^+^ cells. Using another psoriasis model of intradermal injection of recombinant IL-23, we have obtained similar results. Indeed, neutralization of IL-22 in skin with intradermal injection of anti-IL-22 neutralizing antibodies greatly blocked the exacerbation of the skin psoriasis in neoATB mice. These findings altogether support the generality of our conclusions that neonatal antibiotic-treatment could exacerbate the psoriatic inflammation through selective differentiation and/or expansion of IL-22^+^ γδ^+^ T cells, which might also contain IL-22^+^/IL-17^+^ double positive γδ T cells.

Based on data from 16S rRNA analysis we have shown that the antibiotic treatment in neonatal age dysregulated stable formulation and diversity of gut and skin microflora, which surprisingly remained altered till adult age even when the mice were exposed to the normal environment for additional 8 weeks. We have identified particular taxa that could be associated with more exacerbated disease in the neoATB mice. Intriguingly and importantly, cohousing of neoATB mice together with untreated controls suppressed the disease with reduction of the inflammatory cytokines in the skin (significantly decreased production of IL-22 from TCRγδ^+^ cells), likely due to acquisition of ‘healthy' bacteria and correcting the dysbiosis caused by antibiotics. Conversely, transfer of dysregulated microflora from neoATB mice to untreated control mice by cohousing increased severity of disease in cohoused control mice. Moreover, we have examined skin microbiota and observed changes of certain bacterial taxa, suggesting that the changes of skin microbiota might also participate in the differential disease severity between adultATB and neoATB treated mice. For instance, we detected increased abundance of family *Lachnospiraceae* in gut as well as in the skin. Based on these observations, the possibility that neonatal antibiotic treatment may also affect the composition of skin microbiota that influences the development and pathogenesis of psoriatic inflammation cannot be completely excluded. However, as skin is the organ exposed to environment it is difficult to determine, which of the identified species are transient and which species are members of the resident skin community.

We believe that IL-22-producing T cells, especially γδ T cells, play the most important role in the exacerbation of skin inflammation in neoATB mice, whereas down-regulation of IL-22-prodcuing T cells may be an important factor responsible for the improvement/reduction of the skin inflammation in adultATB (although IL-17 producing T cells cannot be completely excluded in the adult treatment model). The different frequencies of IL-22-producing T cells between adultATB and neoATB mice may be attributed to the difference in microbiota in these two groups of mice. Future studies will focus on causative effects of the changes of microbiota on the differential changes of pro-inflammatory T cells, especially TCRγδ^+^ IL-22^+^ T cells. Thus, our findings reveal a previously unrecognized link between imbalances in the microbiota of the gut and the development of experimental psoriasis, which has implications on study of the aetiology and pathogenesis of human psoriasis.

## Methods

### Animals

Mice *C57BL/6* were maintained under specific pathogen-free conditions according to the National Institutes of Health guidelines for the use and care of live animals. All experimental protocols were approved by the Animal Care and Use Committees of National Institute of Dental and Craniofacial Research, NIH.

### Imiquimod model

Mice were dorsally shaved to expose skin. One group was treated with imiquimod cream (Fougera) containing 5% IMQ and the other with control cream (Vaseline) on the ears (5 mg) and on their back (60 mg) over 5–6 days once daily.

### Antibiotic treatment

For ablation of intestinal bacteria in adult mice, an antibiotic cocktail of 500 mg l^−1^ of vancomycin hydrochloride (Hospira) and 100 mg l^−1^ of polymyxin B (X-Gen) were used. Antibiotics were added into the drinking water on 3 days basis. Adult mice (4-weeks-old) were treated with antibiotics for next 4 weeks. Term ‘Neonatal' antibiotic treatment, refers to antibiotic treatment of neonates in interval of 3 weeks, started imediatelly after born. Neonates were treated with combination of both vancomycin (500 mg l^−1^) and polymyxin B (100 mg l^−1^). Topical antibiotic treatment was performed with a combination of vancomycin (500 mg l^−1^) and polymyxin B (100 mg l^−1^) by topical application of antibiotic water on the shaved back skin. Fifteen minutes after topical antibiotic treatment, imiquimod treatment was applied on the dry skin. This treatment was repeated for 5 consecutive days. Vancomycin, an antibiotic that directly targets Gram-positive bacteria, and polymyxin B, an antibiotic that directly targets Gram-negative bacteria, were chosen because both are used in clinical practice and are poorly absorbed when given orally, minimizing the risk of systemic effects on the host. Antibiotic ointment (neosporin) was used for elimination/reduction of skin microbiota. In this particular experiment imiquimod cream was mixed with neosporin (triple antibiotic ointment, 1 g containing: bacitracin 400 units, neomycin 3.5 mg and polymyxin B 5000 units) in the ratio 1:1 (topATB mice) or imiquimod was mixed with 100% pure petroleum jelly in the ratio 1:1 as control treatment.

### Bacterial culture

Skin swabs were obtained from the shaved back skin of mice and soaked in 1 ml of PBS. 1:1 dilutions were prepared and 100 μl of each dilution was spread onto Trypticase soy agar plates with 5% sheep blood (BD). The number of colonies was counted after an overnight incubation in a 37 °C bacterial oven.

### Flow cytometry and reagents

Skin was cleaned of fat tissue and incubated in a Liberase DH (Roche) for 2 h (back skin) or 75 min (ears) before finally shredding through 70-μm and 40-μm cell strainers (BD Pharmingen). Lymph nodes and spleen were mechanically dissociated to yield a single-cell suspension and were treated with ammonium chloride–potassium buffer for red blood cell lysis. After isolation, cell suspensions were passed through 40 μm cells strainers (BD Pharmingen) and cell populations were characterized by flow cytometry. The following antibodies were used for surface staining: anti-mouse TCRβ (H57–597; eBioscience, Catalog numbers: APC eFluor 780 47-5961-82, FITC 11-5961-85, PerCp-Cy5.5 45-5961-82, dilution 1:200), anti-mouse TCRγδ (GL3; eBioscience, Catalog number 11-5711-85, dilution 1:200), anti-mouse CD4 (RM4–5; eBioscience, Catalog numbers: FITC 11-0042-85, PerCp-Cy5.5 45-0042-82, PE-Cyanine7 25-0042-82, dilution 1:200), anti-mouse CD3 (145-2C11; eBioscience, Catalog numbers: APC eFluor 780 47-0032-82, PerCP-Cy5.5 45-0031-82, dilution 1:200), anti-mouse CD45 (30-F11, eBioscience, Catalog number 56-0451-82, dilution 1:200), anti-mouse TCR Vγ2 (UC3–10A6, Biolegend, Catalog number 137706, dilution 1:200), anti-mouse CD194 (CCR4) (2G12, Biolegend, Catalog number 131211, dilution 1:200) and anti-mouse CD196 (CCR6) (29-2L17, Biolegend, Catalog number 129816, dilution 1:200). Dead cells were excluded from analysis using Zombie Yellow Fixable Viability Kit (Biolegend, Catalog number 423104, dilution 1:300). For intracellular cytokine staining, the cells were stimulated for 4 h in phorbol 12-myristate 13-acetate (5 ng ml^−1^) and Ionomycin (1 μg ml^−1^) in the presence of protein transport inhibitor GolgiPlug (BD Pharmingen) containing Brefeldin A (1 μg ml^−1^). Thereafter, cells were surface-stained, washed and fixed using Cytofix/Cytoperm buffer (BD Pharmingen or eBioscience). Intracellular staining was performed with following antibodies: anti-mouse IL-17A (TC11–18H10.1; Biolegend, Catalog number 506916, dilution 1:100), anti-human/mouse IL-22 (IL22JOP; eBioscience, Catalog number 17-7222-82, dilution 1:100) and anti-mouse/rat Foxp3 (FJK-16S; eBioscience, Catalog numbers eFluor 450 48-5773-82, PE 12-5773-82, APC 17-5773-82, dilution 1:100). Neutralizing antibodies for IL-22 (AF582) were purchased from R&D Systems. Samples were analysed with a FACSCalibur and Cellquest software or LSR Fortessa cell analyzer and FACSDiva software (Becton Dickinson). All flow cytometry data were analysed with FlowJo software (TreeStar).

### Intradermal injections

Control or neoATB mice were intradermally injected with recombinant IL-23 (1 μg per day) at opposing sides of shaved back skin (0.5 μg on each side) for 6 consecutive days. For experiments requiring neutralization of local production of IL-22, neoATB mice were intradermally injected at day 1 (50 μg) and day 3 (15 μg) with neutralizing IL-22 antibody (R&D, AF582) during a 4-day imiquimod treatment. Control mice were intradermally injected at day 1 (50 μg) and day 3 (15 μg) with control goat IgG (R&D, AB-108-C) antibody instead.

### Lamina propria lymphocytes preparation

Lamina propria lymphocytes (LPLs) were isolated by mechanical and enzymatic separation from the small intestine and colon. First, segments of the small intestine without Peyer's Patches and the colon were washed extensively and incubated for 20 min at 37 °C with vigorous shaking in pre-warmed RPMI medium supplemented with 3% FBS, 5 mM EDTA, and dithiothreitol (0.145 mg ml^−1^), to remove intraepithelial lymphocyte. Then, LPLs were isolated by further digesting the remaining pieces of tissues with Liberase TL (Roche) (0.2 mg ml^−1^) and 0.05% DNase (Sigma) in RPMI medium for 20 min at 37 °C with continuous stirring. Digested tissues were minced and passed through 70- and 40-μm cell strainer. Lymphocytes were further enriched by Percoll density gradient centrifugation and cells suspensions were re-suspended and characterized by flow cytometry.

### Real-time PCR

Total RNA was extracted using the RNeasy mini and micro kits (Qiagen), following the manufacturer's instructions. The purity of the RNA was assessed by the ratio of absorbance at 260 and 280 nm. RNA purity was within a range of 2.0–2.1. RNA was stored in aliquots at −80 °C until used for reverse transcription. RNA was converted to cDNA using Taq-Man reverse transcription reagents (Applied Biosystems). A reaction mix for real-time PCR was made with Taq-Man Universal PCR master mix, water, and Assays on demand gene expression products for IL-1β, IL-17A, IL-22, IL-23p19, Foxp3 and HPRT (all Applied Biosystems). A measure of 20 μl of reaction mix was aliquoted to the wells on a real-time PCR plate; and each sample was analysed in duplicate. A volume of 5 μl of cDNA was added to each well. The PCR reaction was run on a 7500 real-time PCR System (Applied Biosystems) using standard conditions. Data were analysed using the Multid Genex Software v4.4.2.

### Histology

Skin and ears were collected and fixed in 10% formalin (Richard-Allan Scientific), embedded in paraffin, cut longitudinally into 5-μm sections and stained with haematoxylin and eosin. Images were acquired using Aperio digital pathology system and processed in Aperio ImageScope v11.1.2.752 software.

### Measurement of skin thickness

The thickness of skin was measured by digimatic caliper (Mitutoyo) by at least two investigators and data showed represent average of at least two measurements. Thickness of epidermis was measured using the Aperio ImageScope v11.1.2.752 software.

### 16S rRNA sequencing and analysis

The V4 region of the bacterial 16S rRNA gene was amplified from faecal gDNA. DNA was extracted using Mo Bio PowerSoil DNA isolation kit according manufacturer instructions. Sequencing was performed on the MiSeq platform using a 2 × 250 bp run in multiplex. The QIIME software suite^29^ was used to demultiplex and cluster sequences into 97% identity OTUs and assign taxonomic annotation based on the SILVA v111 SSU database^30^. Analysis and visualization of microbiome communities was conducted in R (ref. 31), using the phyloseq package^32^ to import sample data and calculate alpha- and beta-diversity metrics. Significance of categorical variables were determined using the non-parametric Kruskal–Wallis test^33^. Principal coordinate plots employed the Monte Carlo permutation test^34^ to estimate *P* values.

### Statistics

Statistical significance between control and experimental groups were calculated based upon the number of groups compared. Comparisons were calculated by Student's unpaired *t*-test, if two groups were assessed, or one-way analysis of variance analysis (with Dunnett or Tukey post tests as indicated in figure legends) for more then two group comparisons. Analysis was performed with the GraphPad Prism Software, version 6.

## Additional information

**How to cite this article:** Zanvit, P. *et al*. Antibiotics in neonatal life increase murine susceptibility to experimental psoriasis. *Nat. Commun.* 6:8424 doi: 10.1038/ncomms9424 (2015).

## Supplementary Material

Supplementary Information Supplementary Figures 1-9
