# Supplementary material for: Antibiotics in neonatal life increase murine susceptibility to experimental psoriasis
Source: Nat Commun. 2015 Sep 29;6:8424. doi: 10.1038/ncomms9424 (PMC4598725; doi:10.1038/ncomms9424)
Supplement: Supplementary Information — Supplementary Figures 1-9 [file ncomms9424-s1.pdf]

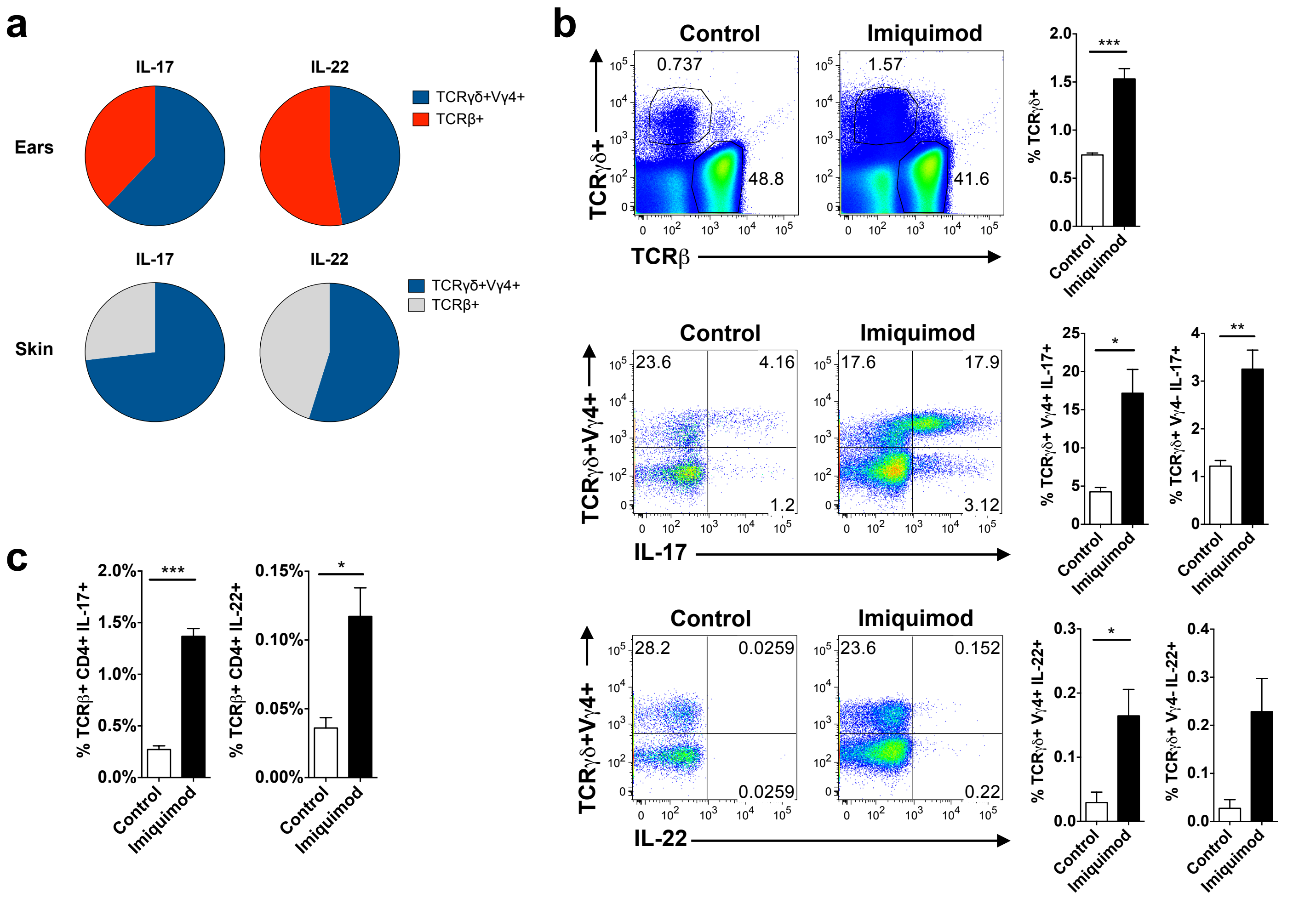

**Supplementary Figure 1**

**Increase of local and systemic inflammation after Imiquimod treatment**

**a)** Total number of IL-17⁺ and IL-22⁺ dermal TCRγδ⁺Vγ4⁺ or TCRβ⁺ cells in back skin and ears of the Imiquimod treated mice. **b)** Representative FACS plot of TCRγδ⁺Vγ4⁺IL-17⁺ or TCRγδ⁺Vγ4⁺IL-22⁺ cells in skin draining lymph nodes after Imiquimod treatment. Control (n=3), Imiquimod (n=4). **c)** Increased frequency of TCRβ⁺CD4⁺IL-17⁺ and TCRβ⁺CD4⁺IL-22⁺ cells in spleen after Imiquimod treatment. Control (n=3), Imiquimod (n=4). Data representative of 3 experiments. Results are shown as mean ± S.E.M., significance was determined by unpaired two-tailed student's *t*-test (**P* < 0.05, ***P* < 0.01, ****P* < 0.001).

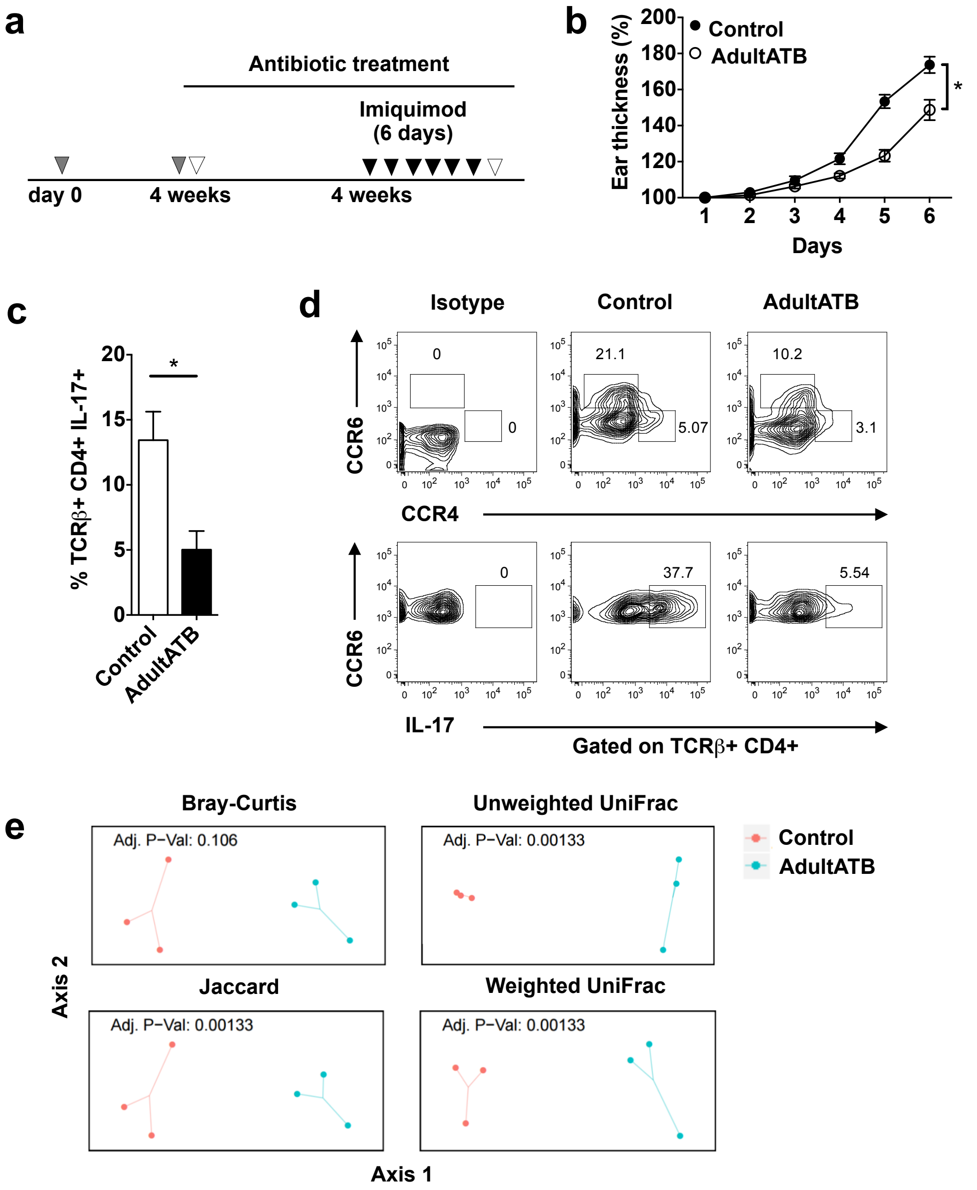


**Supplementary Figure 2**

**Antibiotic treatment in adult mice ameliorates severity of psoriasis induced by Imiquimod**

**a)** Scheme of immunization. **b)** Ear thickness time-course in AdultATB and Control mice during 6-days Imiquimod treatment. **c)** Frequency of TCRβ$^+$CD4$^+$IL-17$^+$ cells in lamina propria lymphocytes (LPL) in small intestine (SI) in AdultATB and Control mice after antibiotic and Imiquimod treatment. Data are representative of 3 experiments. **d)** Effect of antibiotic treatment on the expression of chemokine receptors CCR6 and CCR4 in the LPL-SI after antibiotic treatment. Upper panel: Representative FACS plots of CCR6 vs. CCR4 within CD45$^+$/Live CD4$^+$TCRβ$^+$ cells in the LPL-SI. Lower panel: Frequencies of CCR6$^+$IL-17$^+$ cells within TCRβ$^+$CD4$^+$ T cells in LPL-SI. **e)** Beta diversity (PCoA) was used to compare distance measure (Unweighted and Weighted Unifrac), diversity (Jaccard) or dissimilarity (Bray-Curtis) between AdultATB and Control mice. Results are shown as mean ± S.E.M., significance was determined by unpaired two-tailed student's *t*-test (*$P$ < 0.05).

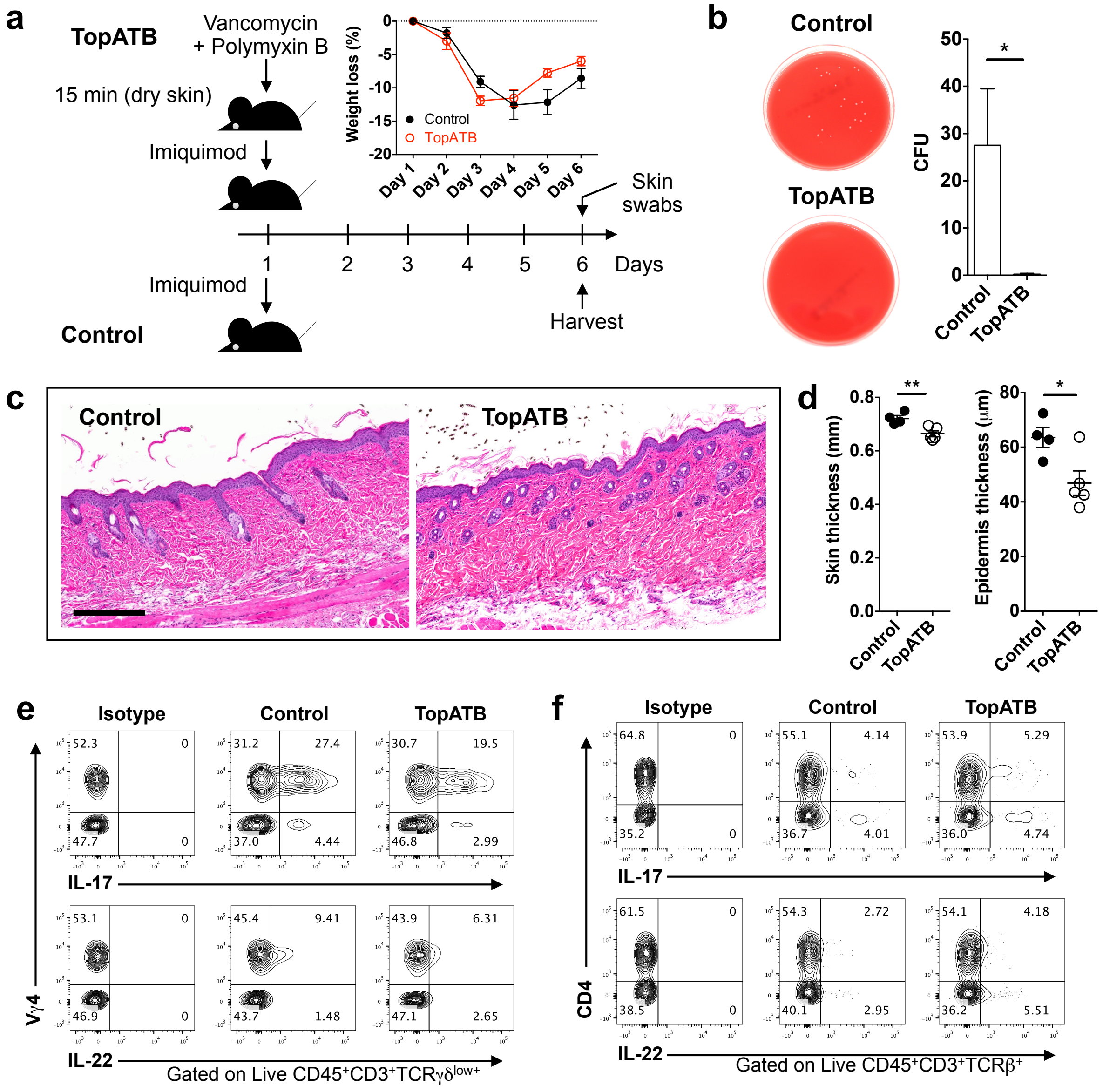


**Supplementary Figure 3**

**Topical antibiotic treatment ameliorates the development of psoriasis induced by Imiquimod with decreased IL-17$^+$ and IL-22$^+$ skin resident γδ T cells.**

**a)** C57BL6 mice (7-weeks old) were treated by topical application of antibiotic water (Vancomycin+Polymyxin B, TopATB, n=5 mice) and after ~15 min the mice were treated on the dry skin with Imiquimod daily for 5 consecutive days. Control mice (n=4) were treated daily only with Imiquimod for 5 consecutive days. Mice were monitored daily; skin tissues were harvested at day 6 (see scheme on A). **b)** Skin swabs were collected at day 6 after treatment and were soaked in PBS. An aliquot of eluted skin bacteria was spread on 5% sheep blood agar plates and cultured overnight at 37ºC. 16h later, the number of Colony Forming Units (CFU) was calculated. Controls n=4, TopATB n=5. **c)** H&E analysis of skin sections of Control and TopATB mice. Scale bar = 300μm. **d)** Thickness of skin measured by Digimatic Caliper at day 6 in Control and Imiquimod treated mice. Data are shown as mean ± S.E.M. of the skin thickness of individual mice. Epidermal thickness was measured using ImageScope Software. **e)** Representative FACS plots of Vγ4$^+$TCRγδ$^+$IL-17$^+$ or Vγ4$^+$TCRγδ$^+$IL-22$^+$ cells in the skin of Control or TopATB mice. **f)** Representative FACS plots of TCRβ$^+$CD4$^+$17$^+$ or TCRβ$^+$CD4$^+$IL-22$^+$ cells in the skin of Control or TopATB mice. Results are shown as mean ± S.E.M., significance was determined by unpaired two-tailed student's *t*-test (*$P$ < 0.05, **$P$ < 0.01).

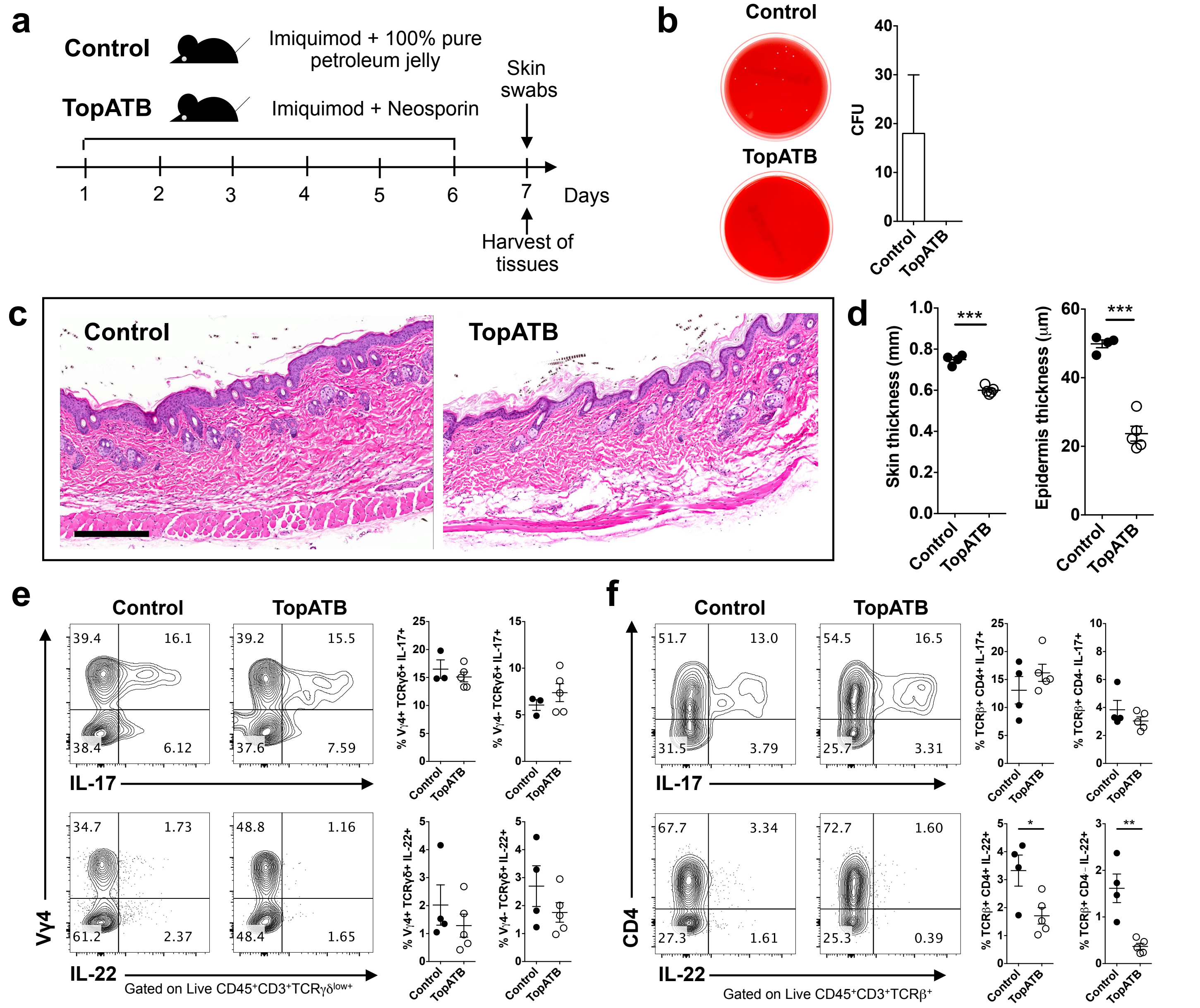


**Supplementary Figure 4**

**Topical treatment with an antibiotic ointment ameliorates development of Imiquimod-induced psoriasis**

Immunization scheme. Mice were treated daily by either topical application of Imiquimod or 100% pure petroleum jelly (Control, n=4), or with a combination of Imiquimod and antibiotic ointment - Neosporin (TopATB, n=5) for 6 consecutive days. **b)** Skin swabs were collected at day 7 after treatment and were soaked in PBS. An aliquot of eluted skin bacteria was spread on 5% sheep blood agar plates and cultured overnight at 37ºC. 16h later, the number of Colony Forming Units (CFU) was calculated. Control n=4, TopATB n=5. **c)** Representative H&E staining of skin samples collected at day 7. Scale bar = 300µm. Thickness of skin was measured using Digimatic Caliper at day 7 in Control and TopATB treated mice. Data are shown as mean ±S.E.M. of the skin thickness of individual mice. Epidermal thickness was measured using ImageScope Software. **d)** Representative FACS plots of $V\gamma4^{+}$IL-$17^{+}$ (upper panel) or $V\gamma4^{+}$IL-$22^{+}$ cells (lower panel) in skin of Control (n=3-4) or TopATB mice (n=5). **e)** Representative FACS plots of $TCR\beta^{+}$CD4+IL-$17^{+}$ (upper panel) or $TCR\beta^{+}CD4^{+}$IL-$22^{+}$ cells (lower panel) in skin of Control (n=4) or TopATB mice (n=5). Results are shown as mean ± S.E.M., significance was determined using an unpaired two-tailed student's *t*-test (**P* < 0.05; ***P* < 0.01).

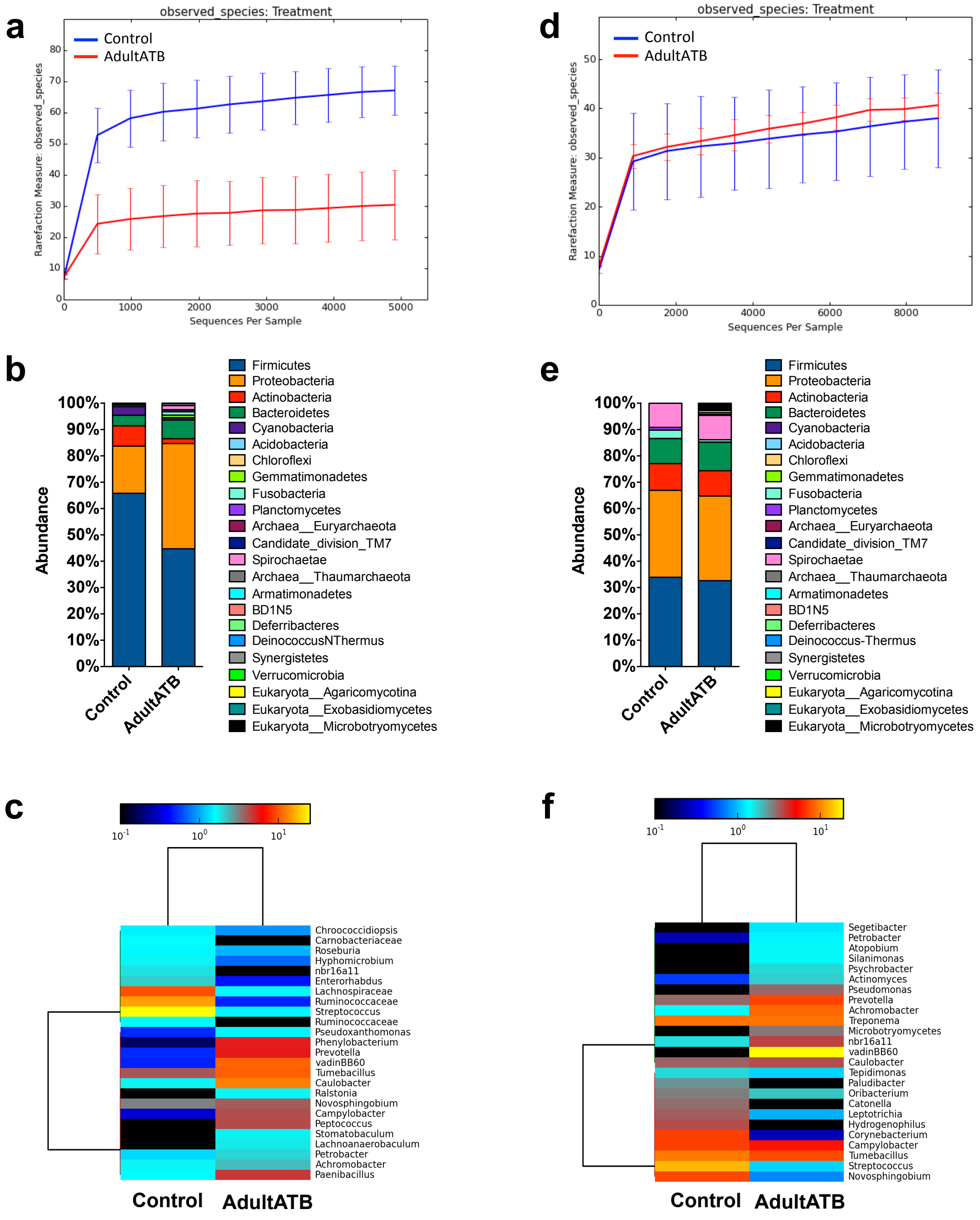


**Supplementary Figure 5**

**16S rRNA analysis of skin microflora before and after Imiquimod treatment in mice exposed to antibiotics at adult age**

Skin swabs from the shaved back skin of AdultATB or Control mice were collected (n=3) and analyzed using 16S rRNA sequencing of region V4. **a)** Diversity of the skin microflora (Observed Species) in AdultATB treated mice compared to Controls before Imiquimod treatment in adult age. **b)** Skin microflora analysis at the phylum level between AdultATB and Control mice before Imiquimod treatment in adult age. Summarizing data showing average from 3 mice **c)** Heat map analysis showing top 25 different skin taxa in the skin of AdultATB and Control mice before Imiquimod treatment. **d)** Diversity of the skin microflora (Observed Species) in AdultATB treated mice compared to Controls after Imiquimod treatment in adult age. **e)** Skin microflora analysis at the phylum level between AdultATB and Control mice after Imiquimod treatment in adult age. Summarizing data showing average from 3 mice. **f)** Heat map analysis showing top 25 different skin taxa in the skin of AdultATB and Control mice after Imiquimod treatment.

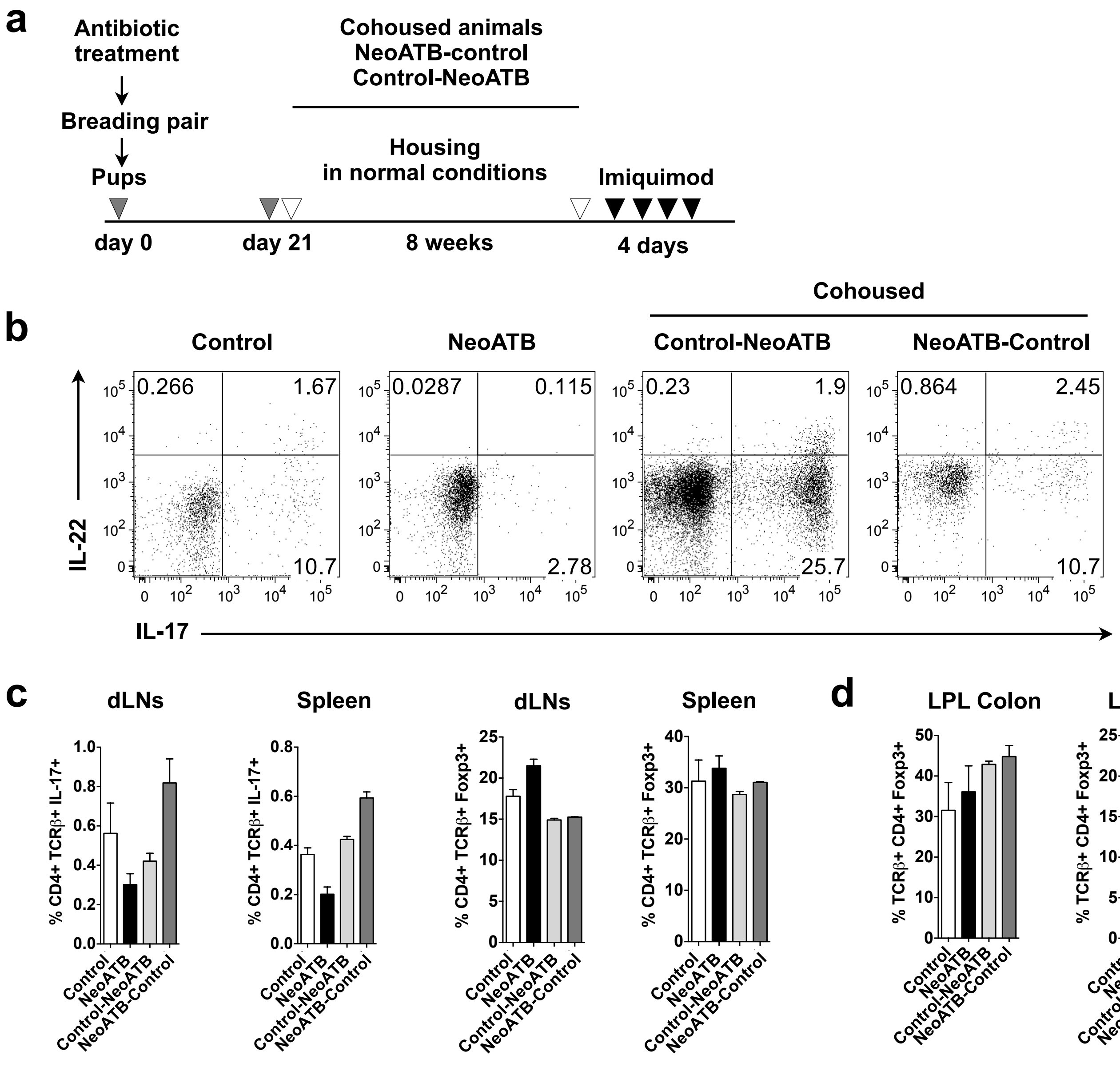


**Supplementary Figure 6**

**Neonatal antibiotic treatment increases severity of psoriasiform dermatitis induced by Imiquimod treatment in adult age**

**a)** Immunization scheme **b)** Representative FACS plots of frequencies of Th17 cells within lamina propria lymphocytes in small intestines of Control, NeoATB, and co-housed (Control-NeoATB and NeoATB-Control) mice after Imiquimod treatment. Cells were gated on $CD45^{+}TCR\beta^{+}CD4^{+}$ cells. Data are representative of 3 experiments. **c)** Frequency of $TCR\beta^{+}CD4^{+}IL\text{-}17^{+}$ and $TCR\beta^{+}CD4^{+}Foxp3^{+}$ cells in the skin draining lymph nodes (dLN) and spleen after Imiquimod treatment in NeoATB, Control, Control-NeoATB and NeoATB-Control groups. Results are shown as mean ± S.E.M. **d)** Frequency of $TCRb^{+}CD4^{+}Foxp3^{+}$ cells in the lamina propria lymphocytes (LPL) of colon or small intestine (SI) after Imiquimod treatment in NeoATB, Control, Control-NeoATB and NeoATB-Control groups. Results are shown as mean ± S.E.M.

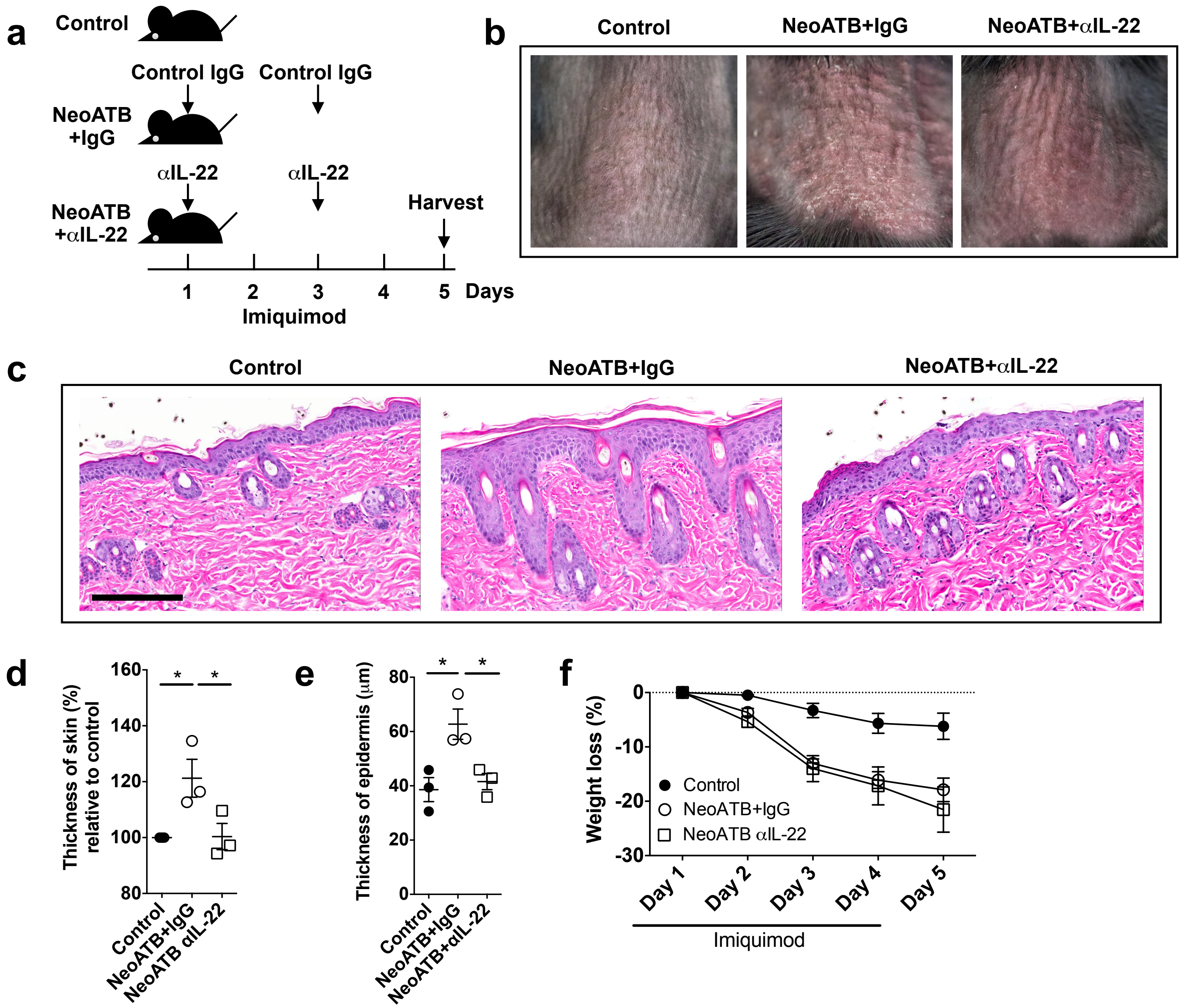


**Supplementary Figure 7**

**Intradermal injection of anti-IL-22 antibody in NeoATB mice ameliorates severity of disease induced by Imiquimod**

**a)** Experimental scheme. NeoATB mice (n=3) were intradermally injected at day 1 (50 μg) and day 3 (15 μg) of control IgG (NeoATB+IgG) or neutralizing anti-IL-22 (NeoATB+aIL-22) antibodies during 4 days of Imiquimod treatment. Mice without neonatal antibiotics treatment (Control, n=3) were treated only with Imiquimod for 4 days. **b)** Representative photographs of back skin in Control, NeoATB+IgG and NeoATB+aIL-22 injected mice 4 days after Imiquimod treatment. **c)** Representative H&E staining of skin samples of Control, NeoATB+IgG and NeoATB+aIL-22 injected mice collected at day 5. Scale bar = 200μm. **d)** Skin thickness of Control, NeoATB+IgG or NeoATB+aIL-22 treated mice measured at day 5. Skin thickness was measured by digimatic caliper at least by two measurements and data are shown as mean ± S.E.M. of the measurements of individual mice. **e)** Epidermal thickness was measured using ImageScope Software. **f)** Weight loss of Control, NeoATB+IgG or NeoATB+aIL-22 injected mice during Imiquimod treatment monitored daily. Results are shown as mean ± S.E.M., significance was determined using one-way ANOVA followed by Tukey's posttest (*$P < 0.05$).

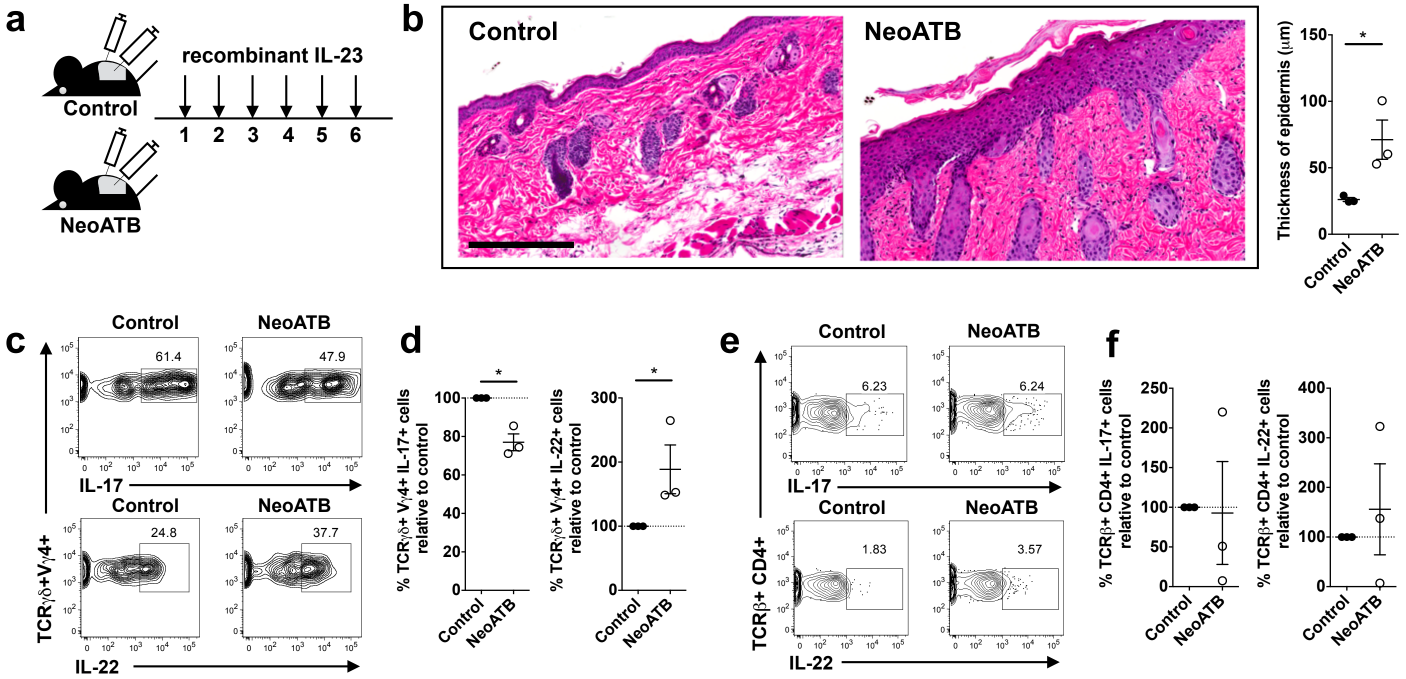


**Supplementary Figure 8**

**Neonatal antibiotic treated mice developed an exacerbated form of psoriasis after recombinant IL-23 injection at adult age**

**a)** Immunization scheme. Control or NeoATB mice were intradermally injected with recombinant IL-23 (1 μg/day) in two locations (0.5 μg to each) on either side of shaved back skin for 6 consecutive days **b)** Haematoxylin and eosin (H&E) staining of back skin at day 6 after daily injection of recombinant IL-23, Scale bar = 200μm. The bar graph indicates the measurements of thickness of epidermis. Thickness of epidermis was determined using ImageScope software along different areas of epidermal layer. **c)** Flow cytometry of $TCR\gamma\delta^{+}V\gamma4^{+}IL\text{-}17^{+}$ (upper panel) and $TCR\gamma\delta^{+}V\gamma4^{+}IL\text{-}22^{+}$ (lower panel) cells in the skin of control and NeoATB treated mice after 6 consecutive IL-23 intradermal injections. **d)** Relative frequencies of $TCR\gamma\delta^{+}V\gamma4^{+}IL\text{-}17^{+}$ and $TCR\gamma\delta^{+}V\gamma4^{+}IL\text{-}22^{+}$ cells normalized to frequencies in control mice (*$P$ < 0.05). **e)** Flow cytometry of $TCR\beta^{+}CD4^{+}IL\text{-}17^{+}$ (upper panel) and $TCR\beta^{+}CD4^{+}IL\text{-}22^{+}$ cells in the skin of control and NeoATB treated mice after 6 consecutive IL-23 intradermal injections. **f)** Relative frequencies of $TCR\beta^{+}CD4^{+}IL\text{-}17^{+}$ and $TCR\beta^{+}CD4^{+}IL\text{-}22^{+}$ cells normalized to frequencies in control mice. Data represents summary of 2 independent experiments. Data were analyzed using unpaired student *t*-test (*$P$ < 0.05).

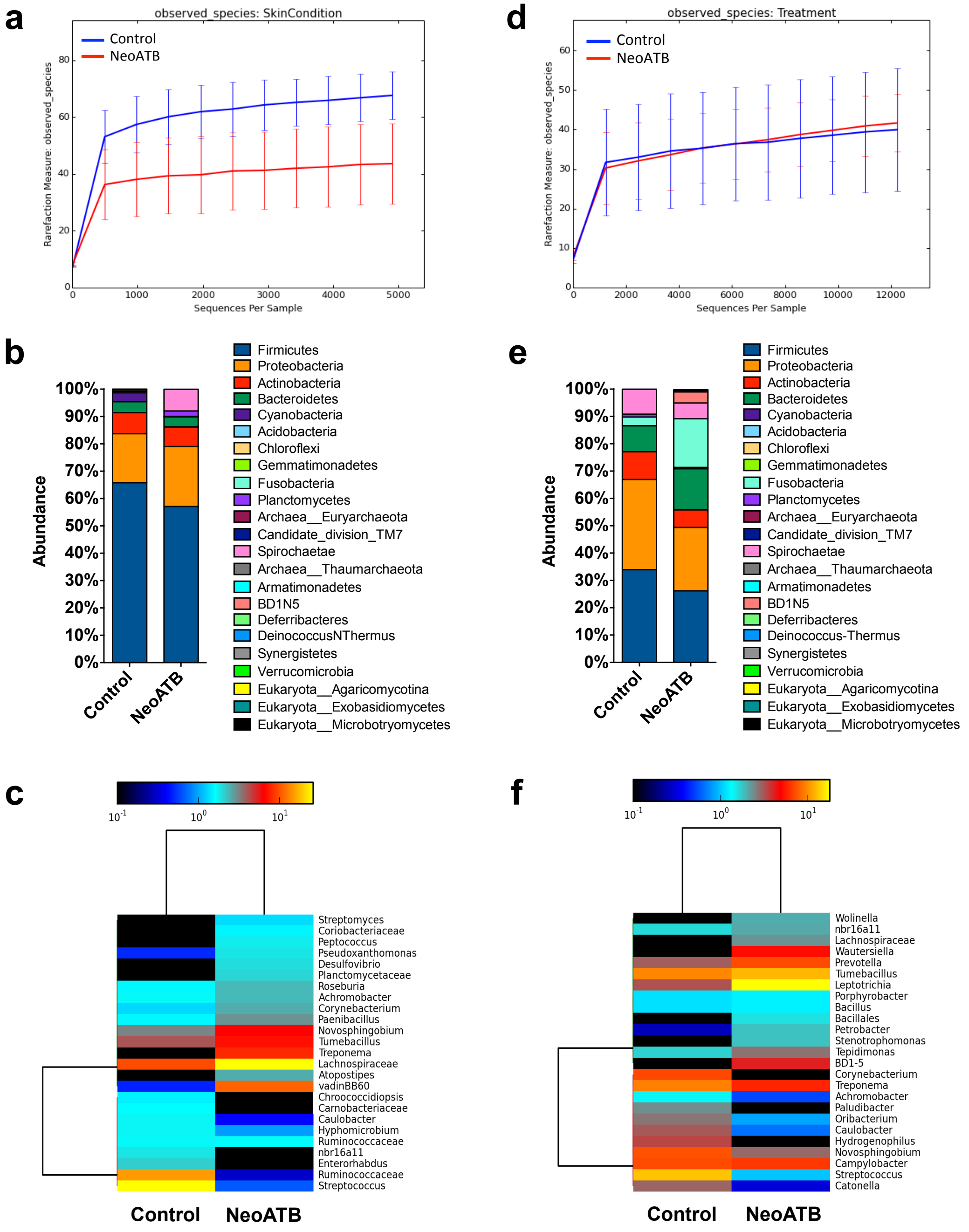


**Supplementary Figure 9**

**16S rRNA analysis of skin microflora before and after Imiquimod treatment in mice exposed to antibiotics at neonatal age**

Skin swabs from the shaved back skin of NeoATB or Control mice were collected (n=3) and analyzed using 16S rRNA sequencing of region V4. **a)** Diversity of the skin microflora (Observed Species) in NeoATB treated mice compared to Controls before Imiquimod treatment in adult age. **b)** Skin microflora analysis at the phylum level between NeoATB and Control mice before Imiquimod treatment in adult age. Summarizing data showing average of 3 mice **c)** Heat map analysis showing top 25 different skin taxa in the skin of NeoATB and Control mice before Imiquimod treatment. **d)** Diversity of the skin microflora (Observed Species) in NeoATB treated mice compared to Controls after Imiquimod treatment in adult age. **e)** Skin microflora analysis at the phylum level between NeoATB and Control mice after Imiquimod treatment in adult age. Summarizing data showing average of 3 mice **f)** Heat map analysis showing top 25 different skin taxa in the skin of NeoATB and Control mice after Imiquimod treatment.
